# Right Vertebral Artery Arising From Ipsilateral Common Carotid Artery With Severe Stenosis of Ostio-proximal Segment of Aberrant Right Subclavian Artery: A Rare Life-Saving Variant

**DOI:** 10.7759/cureus.25566

**Published:** 2022-06-01

**Authors:** Chandrashekar Patil, Sai Kotamraju, Prashanth Kumar, Raja Kollu, Manender Reddy

**Affiliations:** 1 Radiodiagnosis, Malla Reddy Medical College for Women, Hyderabad, IND

**Keywords:** cerebral circulation, variant anatomy, vertebral artery, right brachiocephalic artery, right vertebral artery variant, aberrant right subclavian artery

## Abstract

Anomalous origins of vertebral arteries are rare vascular anomalies that are incidentally identified during computed tomography, magnetic resonance imaging, or digital subtraction angiograms. We present the case of a 45-year-old male who had gangrene of the right ring finger associated with absent radial, ulnar, and brachial artery pulses. A computed tomography angiogram of the upper limb including the arch of aorta showed an aberrant right subclavian artery having near-total stenosis at the origin. An anomalous origin of the right vertebral artery from the right common carotid artery was also noted. This incidental variant anomaly of the vertebral artery was vital in this case as it spared the posterior cerebral circulation from vascular insufficiency complications. It is also important for future head and neck endovascular interventions to avoid inadvertent arterial injury.

## Introduction

The vertebral artery is the main artery of posterior cerebral and cerebellar circulation. It supplies vital areas such as the midbrain, pons, and cerebellum [1]. On each side of the body, the vertebral arteries normally arise from the posterosuperior aspect of the central subclavian arteries, then enter deep into the transverse process. An abnormal origin of the vertebral artery is a rare occurrence [2]. Anomalous origin of vertebral artery from the common carotid artery (CCA) is most commonly associated with an ipsilateral aberrant right subclavian artery (RSCA) from the aortic arch. Here, we present a patient with complaints of blackening of the right third finger over a period of 20 days. On subsequent computed tomographic angiography (CTA), we reported an anomalous origin of the right vertebral artery (RVA) arising from the ipsilateral CCA with severe stenosis of the ostio-proximal segment of the aberrant RSCA. This stenosis was found to be the causative factor for the patient's clinical presentation. However, posterior circulation of the brain was protected by this incidentally detected variant origin of the RVA from the ipsilateral CCA.

## Case presentation

**Figure 1:**
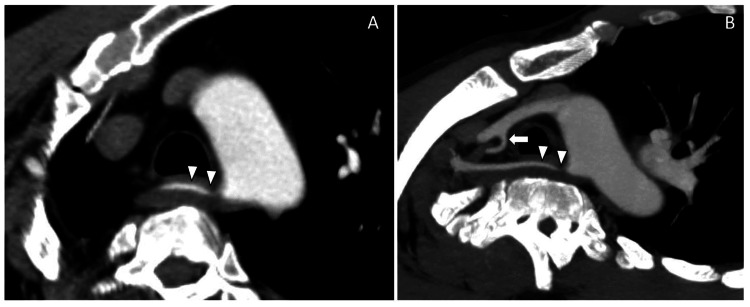
Oblique reformatted thin axial CTA and MIP images showing aberrant RSA origin from the arch of aorta with severe stenosis at the ostio-proximal segment (arrowheads). Note the variant origin of right vertebral artery arising from right common carotid artery (1B, white arrow). CTA: computed tomographic angiography; RSA: right subclavian artery; MIP: maximum intensity projection

**Figure 2:**
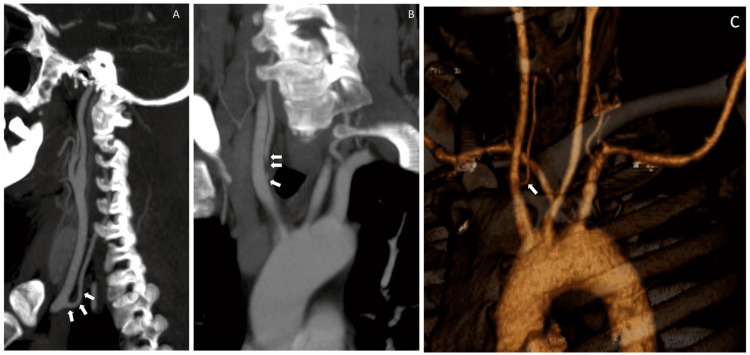
(A) Sagittal MIP, (B) Coronal MIP, and (C) VR reformatted images showing variant vertebral artery arising from common carotid artery (white arrows). MIP: maximum intensity projection; VR: volume rendering

A 45-year-old male presented with complaints of blackening of the right third finger over a period of 20 days. He was hypertensive and not a diabetic. There was no significant family history. He was a chronic smoker and alcoholic. On examination, radial, ulnar, and brachial pulses were not recordable leading to a suspicion of subclavian artery thrombosis. Such a drastic absence of pulses could be due to an occlusion of the RSCA at the origin. However, no significant complaints such as vertigo, nausea, difficulty with balance and ambulation, or difficulty maintaining sitting posture, which are expected as a part of occlusion of corresponding vertebral arteries, were noted. Further investigation with a contrast-enhanced upper limbs CTA was performed, which showed an aberrant RSCA arising from the arch aorta with severe stenosis (approximately 80% stenosis) at the ostio-proximal segment of the RSCA for a length of approximately 25mm (Figures 1A, 1B) with a normal distal flow. A central filling defect was also noted in the right axillary artery causing near-total occlusion with severe attenuation/narrowed caliber of the right brachial artery. Right radial and ulnar arteries are reformed by the collaterals below the level of the elbow joint (not shown here). The RVA was anomalous in origin and was arising from the right common carotid artery (RCCA) (Figures 1B, 2A, 2B, 2C). The patient was managed conservatively and was started on oral antiplatelet and thrombolytic drugs. The majority of the upper limb pulses recovered on follow-up visits, but the gangrenous digit had to be excised to prevent further complications and sepsis.

## Discussion

Atypical vertebral artery origin is known to occur in ~7.0% of the cases [3]. Atypical vertebral artery origins are typically identified incidentally on CTA or preprocedural catheter angiogram studies during endovascular or neuro-interventional procedures. The most widely recognized variety of vertebral artery anomalous origin is the left vertebral artery emerging directly from the aortic arch between the left CCA and left subclavian artery, occurring in approximately 2.4-5.8% of the cases [4]. Of the variant origins of vertebral arteries, more anomalies are noted on the left side elements. The RVA presents less commonly with an aberrant origin (only 0.25% of the cases) [3]. The incidence of the RVA arising from the RCCA and an aberrant RSCA is only ~0.18%. The right and left vertebral arteries usually originate from the posterior superior part of the first part of the subclavian artery, extending medially and cranially into the foramina transversarium of all superior cervical transverse processes. It enters the skull through the atlas and foramen magnum. The growth of the occipital lobe and brainstem is the initial stimulus for the development of the posterior circulation.

Embryologically, the development of the vertebral artery is contributed by the cervical intersegmental arteries, which normally originate from the subclavian artery [5]. Initially, these arteries are the major source of blood supply to the cervical nerves and spinal cord. The development of the vertebral artery starts with the transverse anastomoses between the first six cervical intersegmental arteries, which form the distal vertebral artery. The proximal part of the vertebral artery is formed by the seventh cervical intersegmental artery. The normal origin of the vertebral artery from the subclavian artery requires regression of the aortic end of the first six ventral segments [6]. Anomalies arise when they do not regress. For the anomalous origin of the vertebral artery from CCA to occur, the third, fourth, and fifth cervical intersegmental arteries should not regress [7]. The aberrant RSCA having a retroesophageal course is the last great vessel of the proximal descending aorta.

Thrombosis/occlusion of the RSCA can hamper the posterior cerebral circulation as it forms the main supply for the RVA. Posterior cerebral insufficiency can present with vague and non-specific symptoms such as watershed ischemia symptoms, transient ischaemic attacks, lightheadedness, and altered mental status. Such vague symptoms can delay the time to diagnosis. Posterior cerebral vascular insufficiency, especially vertebrobasilar artery insufficiency, can have an 80-95% mortality rate [8]. Such severe complications and the risk to life were avoided in our case as the posterior cerebral circulation was safeguarded by the variant origin of the RVA from RCCA.

The patient was managed conservatively with oral antiplatelet and thrombolytic drugs. The majority of the upper limb pulses recovered on follow-up visits, but the gangrenous digit had to be excised to prevent further complications and sepsis.

## Conclusions

Knowledge of vertebral artery variants is important as they are asymptomatic and incidentally found; their documentation is important to avoid inadvertent vascular injury during the vascular or neurointervention. In our case, the anomalous origin of the RVA from the patent ipsilateral CCA added clinical advantage to the patient since the ostio-proximal segment of the RSCA was severely stenosed, thereby preserving the vertebral artery circulation. 
